# Antagonistic Changes in Sensitivity to Antifungal Drugs by Mutations of an Important ABC Transporter Gene in a Fungal Pathogen

**DOI:** 10.1371/journal.pone.0011309

**Published:** 2010-06-25

**Authors:** Wenjun Guan, Huifeng Jiang, Xiaoxian Guo, Eugenio Mancera, Lin Xu, Yudong Li, Lars Steinmetz, Yongquan Li, Zhenglong Gu

**Affiliations:** 1 College of Life Sciences, Zhejiang University, Hangzhou, People’s Republic of China; 2 Division of Nutritional Sciences, Cornell University, Ithaca, New York, United States of America; 3 European Molecular Biology Laboratory, Heidelberg, Germany; 4 Department of Molecular Biology and Genetics, Cornell University, Ithaca, New York, United States of America; The Research Institute for Children at Children's Hospital New Orleans, United States of America

## Abstract

Fungal pathogens can be lethal, especially among immunocompromised populations, such as patients with AIDS and recipients of tissue transplantation or chemotherapy. Prolonged usage of antifungal reagents can lead to drug resistance and treatment failure. Understanding mechanisms that underlie drug resistance by pathogenic microorganisms is thus vital for dealing with this emerging issue. In this study, we show that dramatic sequence changes in *PDR5*, an ABC (ATP-binding cassette) efflux transporter protein gene in an opportunistic fungal pathogen, caused the organism to become hypersensitive to azole, a widely used antifungal drug. Surprisingly, the same mutations conferred growth advantages to the organism on polyenes, which are also commonly used antimycotics. Our results indicate that Pdr5p might be important for ergosterol homeostasis. The observed remarkable sequence divergence in the *PDR5* gene in yeast strain YJM789 may represent an interesting case of adaptive loss of gene function with significant clinical implications.

## Introduction

Opportunistic fungal infections are a global health threat [1]. Widespread use of antifungal drugs in the immunocompromised population has been associated with the emergence of clinically significant drug resistance among patients who have been exposed to such antimycotics for prolonged periods [2], [3]. Understanding the processes that underlie the emergence of such resistance is vital for dealing with this critical issue. Known molecular mechanisms of drug resistance in fungi include overexpression of genes that encode drug-efflux pumps belonging to the ABC (ATP-binding cassette) family of transporter proteins [4], overexpression or mutation of the target enzyme, and alteration of other enzymes in the same biosynthetic pathway as the target enzyme [5].

The budding yeast, *Saccharomyces cerevisiae*, widely used in baking and ethanol production for industrial usage and human consumption, in general is non-pathogenic. Strain YJM789, however, was derived from a clinical *S. cerevisiae* isolate (YJM128) collected from the lung of an AIDS patient [6], [7]. The YJM789 strain has many phenotypes that are relevant to its pathogenicity, including high-temperature growth [8], pseudohyphae [9], and deadly virulence in mouse models [7], [10]. Its capability of crossing with laboratory strains of S. *cerevisiae* makes it an excellent tool to study genetic systems that underlie these complex phenotypes [8]. As fungal infections are common among immunocompromised individuals, AIDS patients are routinely treated with antifungal drugs in general clinical therapy [11], [12]. Therefore, YJM789 represents an excellent tool for understanding how an organism can survive in antifungal drug environments.

Our initial determination of its genome sequence showed that the *PDR5* gene is highly polymorphic in YJM789 [10]. It has an amino acid difference of 5.3% from the lab strain, S288c, whereas at the whole genome level the difference is only 0.43%. It is particularly noteworthy that most of the amino acid differences occurred in two transmembrane domain regions (TMDRs). In yeast, *PDR5* is an important ABC transporter that actively exports various xenobiotic compounds [13], [14], [15], [16], such as azole antifungal drugs. Loss-of-function mutants for the *PDR5* gene in lab strains show hypersensitivity to a spectrum of antifungal drugs and overexpression of this gene product results in resistance to a variety of chemicals [17], [18], [19], [20]. The mechanism by which Pdr5p recognizes so many structurally and functionally unrelated substrates remains an enigma [21]. Previous studies indicate that the transmembrane domains of the ABC transporters may play major roles in recognizing substrates [22], [23]. Because the hyper-variable regions co-localize with the transmembrane regions of Pdr5p from YJM789, we have investigated the functional consequence of these dramatic changes in this important gene.

In this study, several representative antifungal drugs were used to treat YJM789 and BY4741 (a lab strain that is isogenic with S288c) and the outcomes after drug treatment were compared. In contrast to BY4741, deletion of the *PDR5* gene in YJM789 appeared to have no impact on growth in the presence of both azole and polyene, two antifungal drugs widely used in clinical practice. Interestingly, although YJM789 is hypersensitive to azoles, as expected by loss of Pdr5p-exporter function, it is hyposensitive to polyene antimycotics. Our experiments, using both a laboratory strain (BY4741) and YJM789, show that loss of the *PDR5* gene indeed confers a small, but significant growth advantage to the organism in the presence of polyenes.

## Results

### Dramatic sequence divergence in YJM789 *PDR5* gene

**Figure 1:**
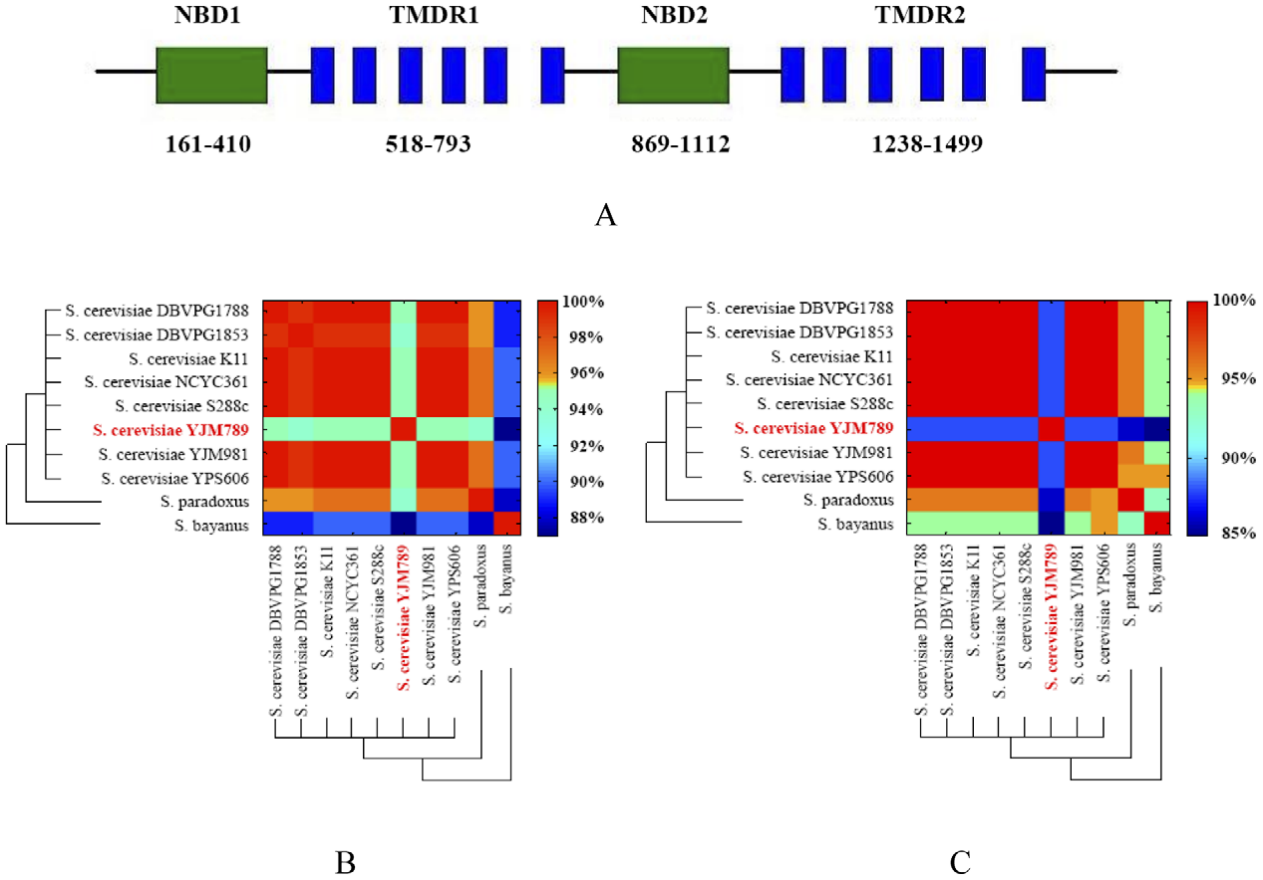
Sequence differences between eight *S. cerevisiae* strains, *S. paradoxus* and *S. bayanus*. A: Schematics of *PDR5* gene regions; B: Amino acid difference for whole *PDR5* sequence; C: Amino acid difference for transmembrane domain regions of *PDR5* sequence. The topology information for the WT Pdr5p was downloaded from UniProtKB/Swiss-Prot database (http://www.uniprot.org/uniprot/P33302). *PDR5* DNA sequences of eight strains of *S. cerevisiae* (DBVPG1788, DBVPG1853, K11, NCYC361, S288c, YJM789, YJM981 and YPS606) were downloaded from a recent study [41]. Only the DNA sequences without any frame-shift mutations were used in this study. DNA sequences of *PDR5* gene in *S. paradoxus* and *S. bayanus* were downloaded from NCBI database. The phylogenetic tree of these species was adapted from Fitzpatrick et al. [60]. The data were analyzed by Matlab and the different color schemes represent levels of amino acid similarity.

Pdr5p belongs to the ABC gene family, a large and important group of proteins that are conserved from bacteria to humans [24]. As shown in Figure 1A, Pdr5p has two transmembrane domain regions (TMDRs) and two nucleotide-binding domains (NBDs). Each TMDR has six stretches of amino acids that span cell membranes and five linkers that connect the transmembrane segments. According to genomic sequences, *PDR5* is a highly polymorphic ORF in YJM789 [10]. Table 1 shows the number of amino acid changes in each part of the gene. Consistent with our previous report, TMD regions have significantly more amino acid changes than do NBD regions in Pdr5p of YMJ789 (chi-square test, *P*<0.0001). Interestingly, within TMDR2, the transmembrane segments also have higher percentage of amino acid changes than the linker regions (Fisher's Exact Test, *P* = 0.04).

**Table 1:** Amino acid changes in each part of *PDR5* gene between YJM789 & S288c (isogenic to BY4741).

	NBD a	TMDR1 b		TMDR2 b	
		TM segments	Linkers	TM segments	Linkers
Length	492	121	155	137	125
Amino acid changes	7	10	12	23	11
% changes	1.42	8.26	7.74	16.8	8.8

a : NBD: Nucleotide Binding Domain.

b : TMDR: Trans-Membrane Domain Region.

To see if *PDR5* is uniquely different in YJM789, we compared the polymorphism and divergence of *PDR5* genes among *S. cerevisiae* strains and other *Saccharomyces* species. Figure 1B and 1C show the pairwise Pdr5p amino acid differences among eight sequenced *S. cerevisiae* strains and *S. paradoxus*, *S. bayanus*, two species in the *Saccharomyces sensu stricto* complex. For the entire Pdr5p sequence, all seven *S. cerevisiae* strains except YJM789 shared more than 99% similarity, but the similarities of YJM789 Pdr5p to those of the other *S. cerevisiae* strains were less than 95% (bright green lane), which are even lower than those between *S. paradoxus* and other *S. cerevisiae* strains (>96%, Figure 1B).

Figure 1C shows pairwise differences for the transmembrane domain regions. The sequence similarities are about 100% among seven *S. cerevisiae* strains except YJM789, which had similarities of only ∼87% to the other strains. In comparison, the average sequence similarities for *S. paradoxus* and *S. bayanus* to other *S. cerevisiae* strains were 96% and 94%, respectively. It is noteworthy also that, except for YJM789, the TMDRs in Pdr5p were more conserved than the rest of the gene within or between species, indicating that TMDRs are more important for gene function. Our results imply that functions of Pdr5p in YJM789 might have been dramatically changed during its evolution.

### YJM789 is hypersensitive to azole antifungal drugs

In the United States, approximately ten antifungal drugs are currently approved by the Food and Drug Administration (FDA) for the therapy of systemic fungal infections. Azoles and polyenes are two principal classes [25]. The azoles can block the ergosterol biosynthesis pathway by inhibiting the enzyme 14-α-demethylase (ERG11), which converts lanosterol to ergosterol and is required in fungal cell membrane synthesis. The polyene antimycotics bind to sterol (ergosterol preferentially) in cell membranes and create holes that lead to ion leakage and fungal death.

**Figure 2:**
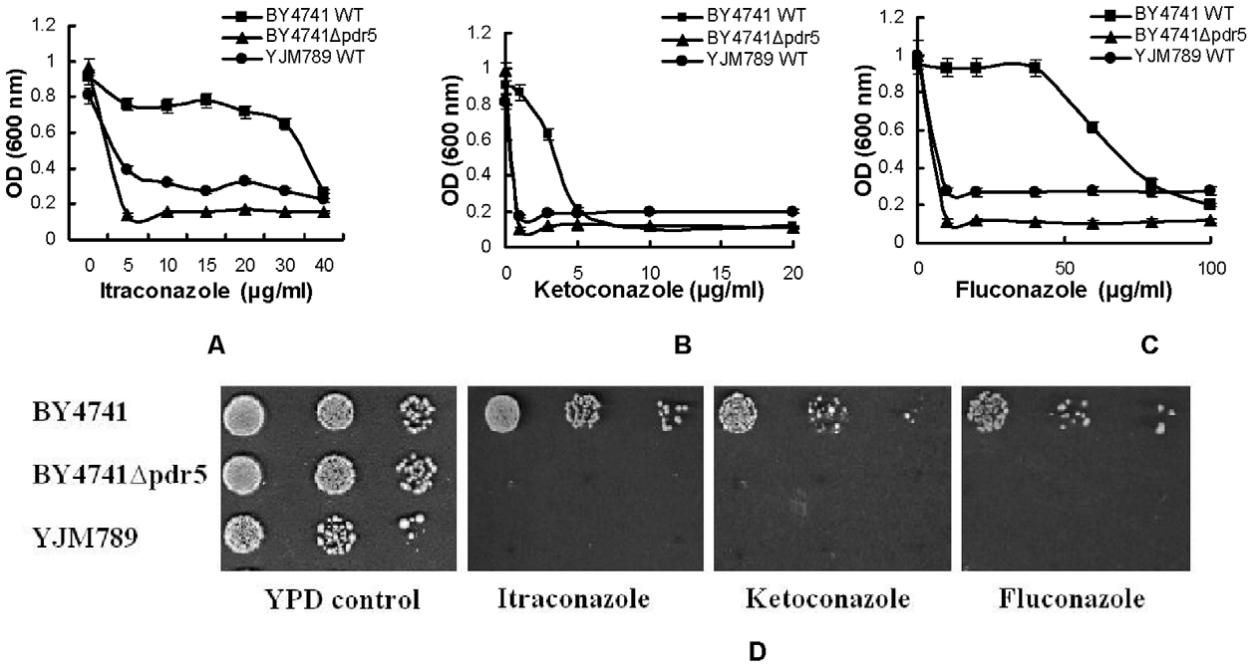
Azole sensitivity of YJM789, BY4741 and BY4741 *PDR5* gene deletion strain. The strains were grown in YPD overnight at 30°C and reinoculated to OD_600_ = 0.1. 90 µL media of strains were treated with 10 µL of water or a pharmacological compound (**A**: itraconazole, **B:** ketoconazole, **C**: fluconazole), respectively, and then grown for 24 h. Only OD_600_ values at 24 h are shown in the figure. Measurements were made in triplicate with standard deviations shown in the figures. **D.** Strains were grown overnight and reinoculated to OD_600_ = 0.2, then 4 µL of ten-fold serial dilutions were spotted onto YPD agar containing one of the drugs (itraconazole: 2 µg/mL, ketoconazole: 1 µg/mL, fluconazole 5 µg/mL), and the plates were incubated at 30°C for 2 days.

**Figure 3:**
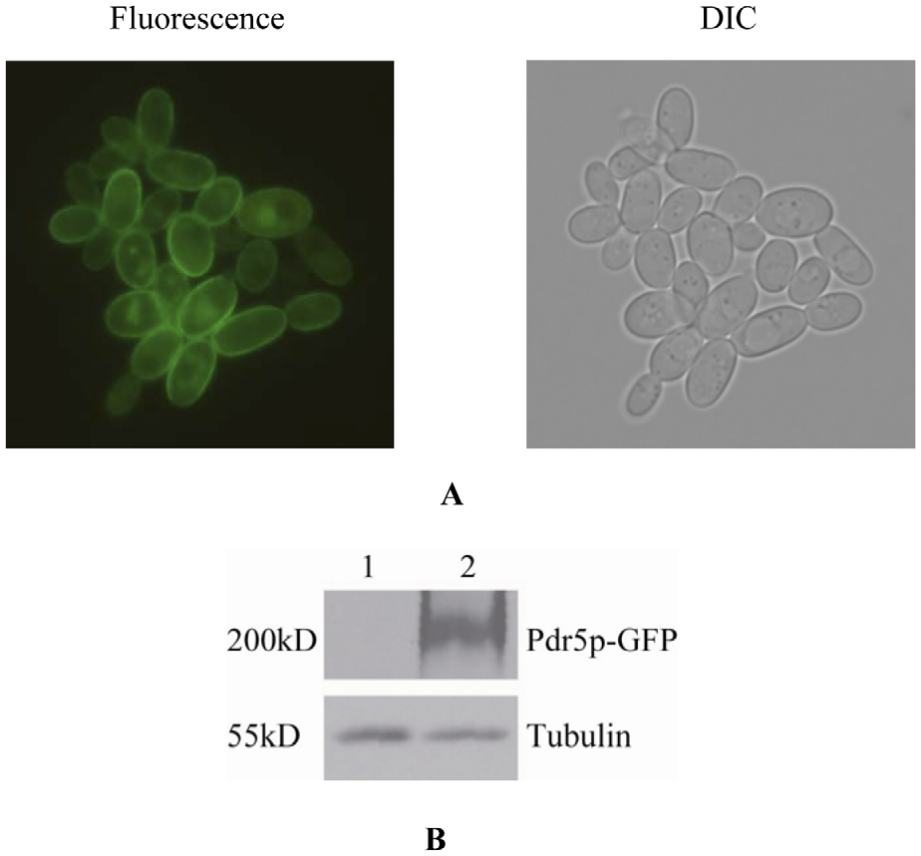
*Pdr5p* localizes and expresses in YJM789. **A**. The strains carrying the GFP-tagged version of Pdr5p were exponentially grown in YPD media and visualized by tagged-GFP signal. Fluorescence (left) and DIC (right) images were background-subtracted and scaled identically. The results clearly show that Pdr5p localizes at plasma membrane in YJM789 strain. **B.** Western blot analysis of Pdr5p (GFP-tagged) in YJM789 strain by using anti-GFP antibody. Lane 1: YJM789 WT, lane 2: YJM789 expressing GFP-tagged Pdr5p. The result indicates that intact Pdr5p could express normally in YJM789 strain.

It is well known that azoles are substrates of Pdr5p in yeast [25], [26]. To investigate the functional consequence of dramatic changes in the YJM789 *PDR5* gene, we compared the growth of YJM789 with that of BY4741 in three selected azoles: itraconazole, ketoconazole, and fluconazole. As shown in Figure 2, YJM789 was much more sensitive than BY4741 to all these drugs. We tagged *PDR5* genes with GFP in both BY4741 and YJM789. Although Pdr5p still localizes in cell membranes and has an intact protein expression (Figure 3), growth patterns of YJM789 were similar to those of the BY4741*Δpdr5* strain when grown in the presence of azoles (Figure 2), which would be expected if Pdr5p drug efflux functions were impaired in YJM789. Considering that YJM789 and BY4741 have significant genetic differences, it is risky to conclude that from this evidence that hypersensitivity of YJM789 to azoles is solely due to its highly divergent *PDR5* gene.

### Azole-efflux function of *PDR5* in YJM789 might be impaired

**Figure 4:**
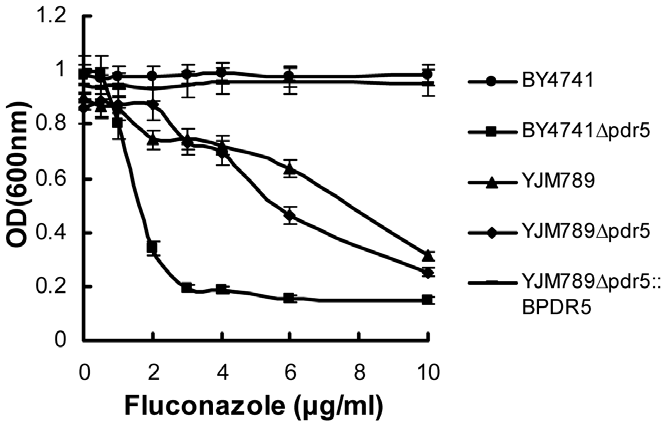
Growth differences between YJM789 and BY4741 in the presence of fluconazole. BY4741, YJM789,their *PDR5* null strains and YJM789*Δpdr5::BPDR5*(GY02) were grown in YPD overnight and reinoculated to OD_600_ = 0.1. 90 µL of the above media with strains were treated with 10 µL of water or different concentrations of fluconazole, and then grown for 24 h at 30°C. OD_600_ values at 24 h are shown in the figure. Measurements were made in triplicate with standard deviations shown in the figures.

To determine if the *PDR5* gene is responsible for the observed drug phenotype in YJM789, we conducted the following two experiments, with fluconazole used to represent azole antimycotics. First, growth profiles of YJM789 were measured before and after the deletion of the *PDR5* gene. If Pdr5p had indispensable drug-exporter function, deletion of the gene would lead to reduced growth of YJM789 in fluconazole. As shown in Figure 4, however, YJM789 and YJM789Δ*pdr5* grew similarly in fluconazole. The fact that Pdr5p played no significant role in YJM789 for resistance to fluconazole suggests that the protein might have lost its ability to export azoles. In the presence of fluconazole, both YJM789 and YJM789Δ*pdr5* grew slightly better than BY4741Δ*pdr5* (Figure 4), indicating that YJM789 may have acquired residual azole-export capability elsewhere in the genome.

**Figure 5:**
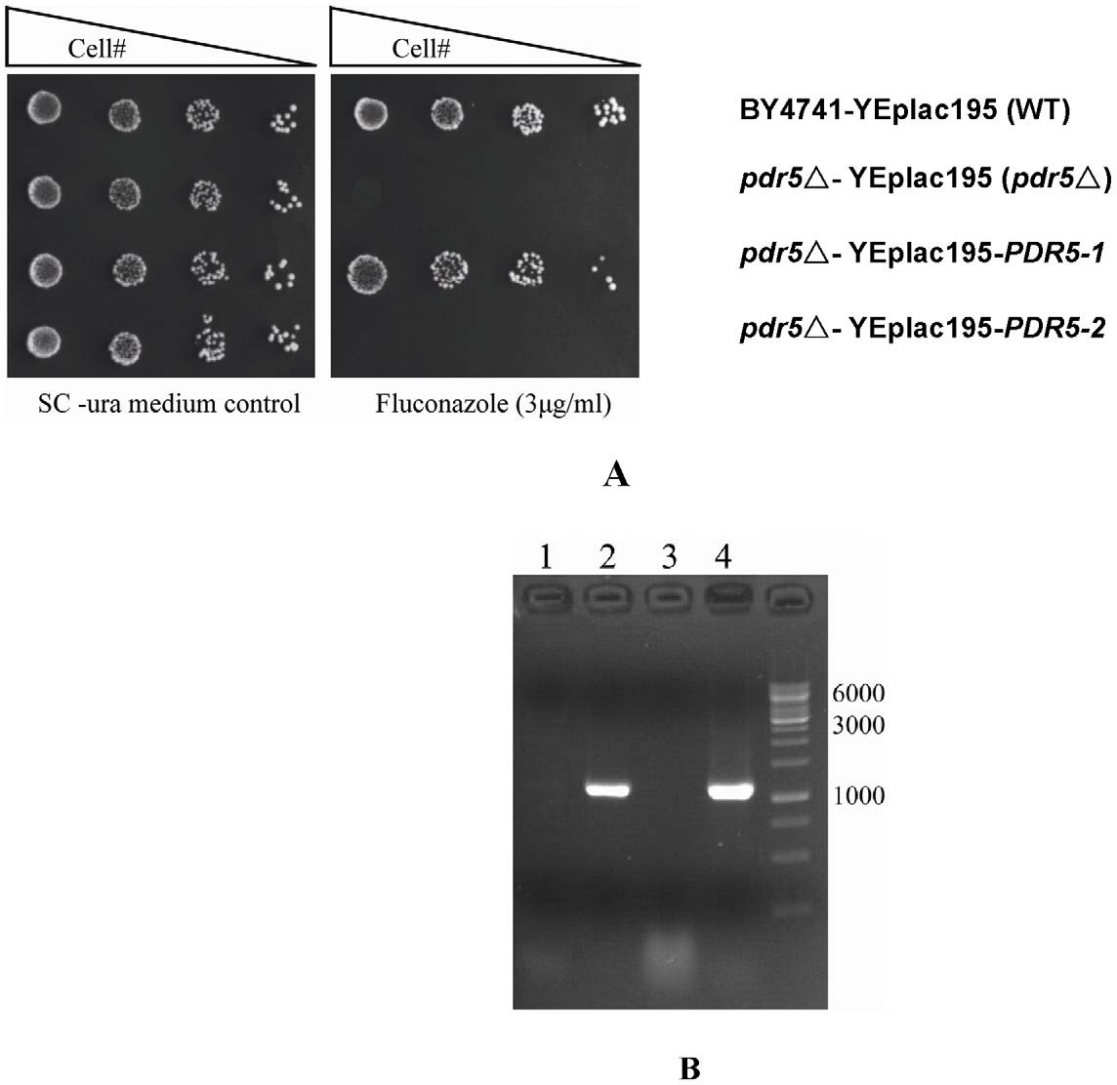
Drug resistance assays with PDR5 variants. **A.** Cell growth on Fluconazole. 4 µl five-fold serial dilutions of BY4741 WT cells, *pdr5* null mutant cells, and cells expressing *PDR5-1*, which was reconstructed with *YPDR5* TMDR1 and *BPDR5* TMDR2 and *PDR5-2*, which was reconstructed with *BPDR5* TMDR1 and *YPDR5* TMDR2, were spotted on SC-uracil drug agar plates. Before spotting, all strains were grown to exponential phase, diluted to 0.2 OD_600_. Plates were incubated for 3 days at 30°C. The results indicate that the TMDR1 in YJM789 *PDR5* is functional (row #1 vs. row #3), but the TMDR2 in YJM789 *PDR5* cannot conduct its original function (row #1 vs. row #4). **B.** Expression of hybrid constructs. Electrophoresis result for RT- PCR products was depicted. Total RNA (lane 1) and cDNA (lane 2) of *pdr5Δ* null mutant cells harboring YEplac195-*PDR5-1*, total RNA (lane 3) and cDNA (lane 4) of *pdr5Δ* null mutant cells harboring YEplac195-*PDR5-2* were amplified by specific primer pairs. The result indicates that both constructs can be expressed successfully in the BY4741Δ*pdr5* background.

Second, to test if BY4741 Pdr5p could accomplish its normal function in the YJM789 background, we replaced the YJM789 *PDR5* gene with the allele from BY4741. The growth profiles for BY4741, YJM789 and YJM789 expressing BY4741 *PDR5* were compared. As shown in Figure 4, YJM789 with BY4741 *PDR5* grew as well as BY4741 WT over the range of fluconazole concentrations, indicating that in the YJM789 background, BY4741 Pdr5p confers normal azole-exporter function. It also indicates that hypersensitivity of YJM789 to azoles was not due to inherent changes in the plasma membrane of YJM789. Our additional results indicated that it is more likely that the TMDR2 domain in the YJM789 *PDR5* gene cannot carry out its original function (Figure 5), which is consistent with the observation that the transmembrane segments in this region have a higher percentage of amino acid changes than other regions (Table 1).

### Mutations in *PDR5* gene lead to gain of resistance to polyene antimycotics

Resistance to antifungal drugs represents a serious health threat. YJM789 is a common yeast strain increasingly isolated from AIDS patients who have received prolonged courses of prophylactic antifungal drug treatment [7]. Because these isolates of YJM789 presumably have survived intensive exposure to antimycotic drugs, particularly the azoles, it was intriguing to observe that YJM789 lost the ability to grow in the presence of antifungal drugs that target ergosterol synthesis.

**Figure 6:**
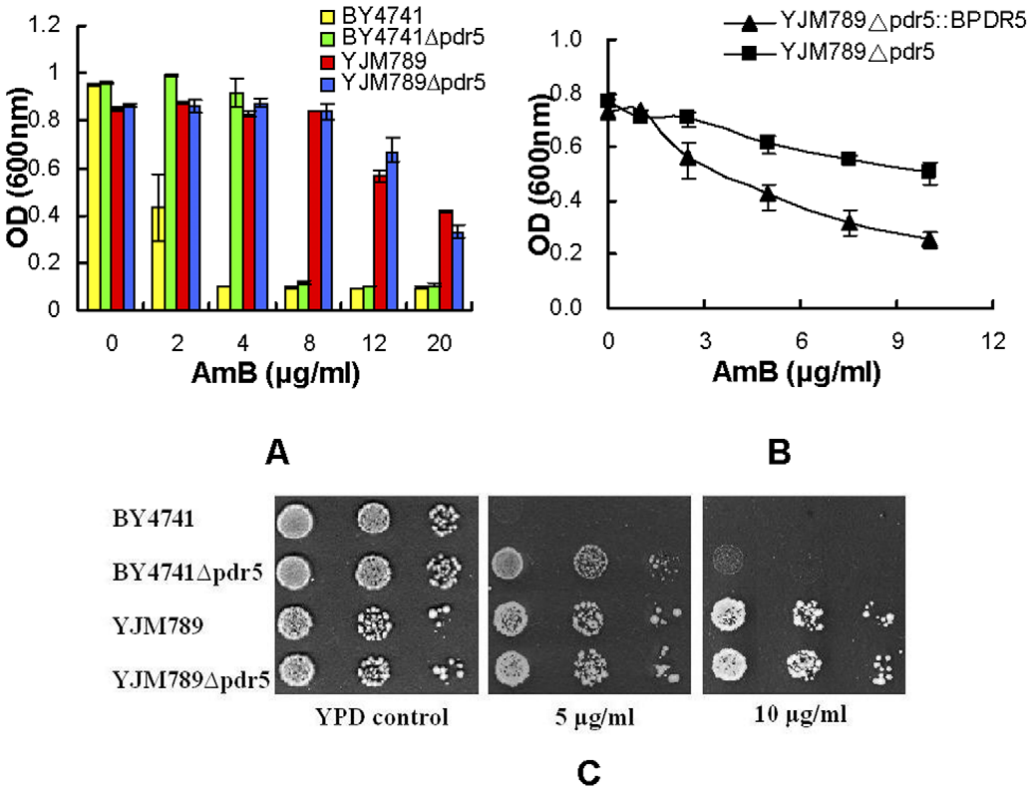
Drug susceptibility of YJM789 and BY4741 in AmB. **A.** All strains were grown in YPD overnight at 30°C and reinoculated to OD_600_ = 0.1. 90 µL of media with strains were treated with 10 µL of water or different concentrations of AmB, and then grown for 20 h at 30°C. The values are the averages from three experiments. **B.** YJM789Δ*pdr5* (GY03) and YJM789Δ*pdr5*:: *BPDR5* (GY02) mutants grew in YPD medium overnight at 30°C and reinoculated to OD_600_ = 0.1. 90 µL of media with strains were treated with 10 µL of water or different concentrations of AmB, and then grown for 20 h at 37°C. Measurements were made in triplicate with standard deviations shown in the figures. **C.** BY4741, YJM789, BY4741Δ*pdr5* and YJM789Δ*pdr5* were grown overnight and reinoculated to OD_600_ = 0.2, then 4 µL of ten-fold serial dilutions were spotted onto YPD agar containing AmB (5 µg/mL, 10 µg/mL), and the plates were incubated at 30°C for 2 days.

To further investigate the reason underlying the high mutations of *PDR5* gene in YJM789, we examined the influence of another group of antifungal drugs: the polyenes. Polyene antimycotics can bind to sterols (ergosterol preferentially) in fungal cell membranes, promoting leakage that contributes to cell death. Amphotericin B (AmB) and nystatin are commonly used polyene antimycotics [27]. Interestingly, YJM789 is much more resistant to AmB (Figure 6A, 6C) and nystatin (data not shown) than is BY4741. Several lines of evidence indicate that mutations in *PDR5* gene of YJM789 are related to its improved growth in this drug environment. First, the BY4741Δ*pdr5* (green bars in Figure 6A) strain was more resistant to AmB than BY4741WT (yellow bars in Figure 6A), indicating that loss of *PDR5* gene function led to a growth advantage in the presence of AmB. Second, strain YJM789 showed similar growth profiles both before and after *PDR5* deletion in the presence of AmB (Figure 6A, 6C). Third, and most importantly, YJM789 strains containing a functional BY4741 *PDR5* gene became more sensitive to AmB than did YJM789 strains without a functional *PDR5* gene (Figure 6B). Interestingly, this growth difference was significant only at 37°C, the temperature of the environment from which YJM789 was isolated, whereas it was difficult to see this difference at 30°C. Of note, YJM789 with a functional BY4741 *PDR5* gene showed a growth advantage over wild-type YJM789 in the presence of fluconazole at both at 30°C (Figure 4) and 37°C (Figure S1).

## Discussion

Genetic variation among individuals contributes to the fitness landscape of a population. Loss of gene function can have dramatic fitness consequences for individuals. Can loss of a gene be adaptive? The antagonistic pleiotropy hypothesis states that certain genes, when functional, are beneficial in some conditions and deleterious in others [28], [29], [30], [31]. This genetic pleiotropic effect is a result of interactions between genes and environments, which can lead to a trade off for organismal fitness under certain conditions. When environments change, the antagonistic pleiotropy of gene functions can lead to adaptive gene loss in evolution. Indeed, this “less is more” scenario was proposed as one model for phenotypic evolution [32], [33]. Accumulating evidence for adaptive gene loss indicates that antagonistic pleiotropy of gene function may play an important role in species adaptation [34], [35], [36], [37].

In this study, we discovered an interesting case of possible adaptive functional loss that has clinical relevance. Our data show that the *PDR5* gene in YJM789 has lost the ability to facilitate growth in the presence of azoles, presumably due to the inability to recognize and export members of this class of antifungal drug. It has been reported that mutations in *PDR5* gene in YJM789 may be responsible for its hypersensitivity to cycloheximide [38]. We showed that deletion of *PDR5* gene, however, improves organism growth in the presence of polyenes. Interestingly, among recently genotyped *S. cerevisiae* strains [39], several different pathogenic *S. cerevisiae* isolates showed large sequence divergence in the *PDR5* gene, which was not observed in any non-pathogenic strains, indicating that dramatic changes in this gene could be important for the pathogenicity of *S. cerevisiae* (Figure S2). We are currently conducting experiments to address this issue in these pathogenic yeast strains.

Because at least two azole drugs were brought to market (ketoconazole and miconazole) before the isolation of YJM789 and other clinical strains [6], it is intriguing to observe that a yeast strain with high azole sensitivity could still survive in the pathogenic *S. cerevisiae* population. One possibility is that the patients from whom the strains were isolated didn't receive intensive treatment with azole antifungal drugs. However, if the gene has been in the population for a considerable time, it seems less likely that azoles were not used extensively in this population. YJM789 is a haploid derivative of the heterozygous diploid clinical isolate YJM128 [7]. The highly mutated allele we observed could be protected by another copy due to the rare occurrence of meiosis in these clinical strains. Indeed, both the complicated life history of pathogenic yeast strains and adaptation to antifungal drugs (polyenes) might function together to keep this highly mutated *PDR5* allele at a certain frequency in pathogenic population of *S. cerevisiae*.

**Figure 7:**
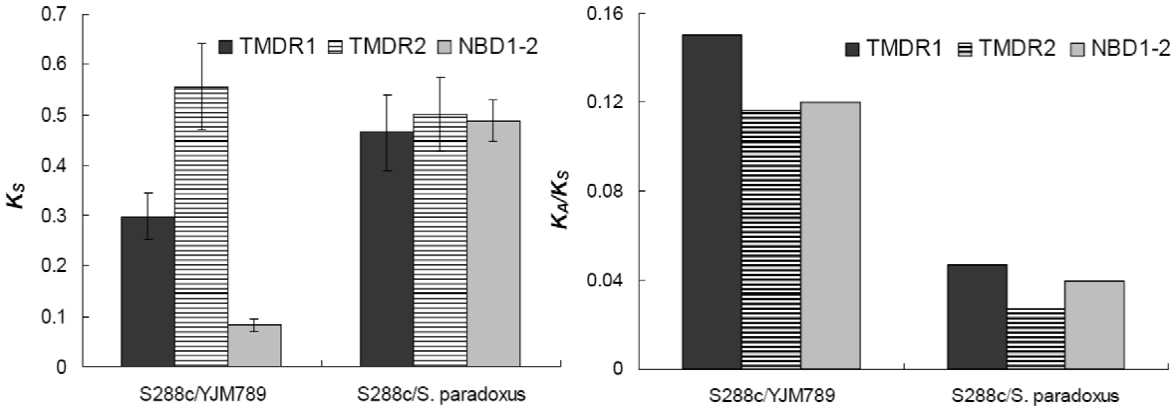
Synonymous evolutionary distances (A) and ratio of non-synonymous to synonymous evolutionary distances (B) in different parts of *PDR5* gene. The distances were calculated using PAML [57]. The bars to the left indicate the distances (ratio) between S288c and YJM789 while the bars to the right measure the distances (ratio) between S288c and *S. paradoxus*.

The origin of the highly polymorphic regions in the YJM789 *PDR5* gene is not clear. The synonymous distance (*K_S_*) of two transmembrane domain regions between S288c and YJM789 are 0.3 and 0.5, respectively (Figure 7A), which are similar to the synonymous distance between S288c and *S. paradoxus*, another *Saccharomyces* species, whereas *K_S_* for the rest of the gene between S288c and YJM789 is 0.071, similar to the genome average [10]. AmB, a polyene antimycotic, was made available in the early 1960s, and is widely used in human immunodeficiency virus (HIV)-seropositive patients [40]. Since antifungal drug therapy has a short history, we have to emphasize that adaptation to polyenes by modification of the *PDR5* gene, on the assumption that it did occur, likely represents an evolutionary force that selected a pre-existing genetic variation.

The closest ABC-family member to the *PDR5* gene in *S. cerevisiae* genome (PDR15 gene) has a 24% amino acid difference from Pdr5p and the synonymous differences between *PDR5* and *PDR15* genes are saturated (*Ks*>5), indicating that the two TMDRs in the YJM789 *PDR5* gene could not have originated from ectopic gene conversion within the same species. A phylogenetic tree was built for the two TMD regions within orthologs of Pdr5p for all sequenced strains of *S. cerevisiae* and *S. paradoxus* [41]. As shown in Figure S3, TMD regions in Pdr5p from YJM789 have the most divergent sequences among strains of *S. cerevisiae*, which is not the case for other genes in the YJM789 genome [41]. Furthermore, TMDRs in the YJM789 *PDR5* gene also show great sequence divergence from strains of *S. paradoxus*, indicating that these two domains were not from *S. paradoxus*, or at least from strains of that species with available genome sequences. No matter what are the origins of these two TMDRs in the YJM789 *PDR5* gene, the ratio of nonsynonymous to synonymous changes across the whole *PDR5* gene significantly increased in YJM789 (Figure 7B), indicating functional relaxation of this gene, which is consistent with our experimental data for inactivation of important functions in Pdr5p of strain YJM789.

The observation of a gain in AmB resistance as a result of the loss of *PDR5* gene is surprising. Ergosterol is an important component of fungal membranes and serves the same function as cholesterol in animal cells. When ergosterol content decreases in fungal plasma membranes, AmB-binding sites might decrease, leading to AmB resistance [42], [43]. If this is indeed the mechanism for AmB resistance after *PDR5* deletion, our results imply that Pdr5p is involved in ergosterol homeostasis. Consistent with our speculation, some studies have reported that Pdr5p may transport lipids, such as phospholipids [44], sphingolipids [45] or glycerophospholipids [46], although the mechanisms of transport are unclear. There are no reports of ergosterol transport by Pdr5p. Further experiments are needed to elucidate the functional role of Pdr5p in ergosterol homeostasis.

It is important to point out that the *PDR5* gene in YJM789 might not be correctly called a pseudogene. The fact that Pdr5p could localize in cell membranes and has an intact protein expression in YJM789 indicates that the pleiotropic protein Pdr5p might conduct unrecognized functions. Because Pdr5p is a multi-substrate transporter [47], our observation could be caused by changes in substrate specificity as a result of mutations in the Pdr5p TMDRs of YJM789. Indeed, this conclusion is supported by the observation that the ratio of non-synonymous to synonymous distances between S288c and YJM789 is less than 0.2 for the *PDR5* gene (Figure 7B).

It is also important to note that other genetic changes contribute to the YJM789 AmB-resistance phenotype. At higher concentrations of AmB, growth of BY4741 and BY4741Δ*pdr5* was inhibited, whereas YJM789 and YJM789Δ*pdr5* still showed significant growth, indicating that changes in other genes in YJM789 contributed to AmB adaptation. It was shown that mutations in ERG6, an important gene in the ergosterol biosynthesis pathway, can lead to AmB resistance in various pathogenic species of yeast [48], [49]. Our analysis indicated that ERG6 gene and its ∼300 bp 5′ UTR region are identical in BY4741 and YJM789, indicating that the gene is not likely involved in AmB resistance of YJM789. By using a genotyped progeny panel (∼200 strains) from a cross between BY and YJM789 [50], we are currently conducting QTL-mapping to identify genetic loci that underlie this interesting phenotype.

Regardless of the evolutionary history of the *PDR5* gene and the mechanism for the AmB-sensitivity change in YJM789, our experimental results might imply a new drug-resistance strategy in pathogenic yeasts, *i.e.*, sacrificing an important function in one drug condition for a minor fitness gain in other drug conditions. This clinically important showcase with interesting evolutionary implication will, hopefully, lead to better understanding of the emergence of drug resistance not only in pathogenic fungi, but also in microbes in general.

## Materials and Methods

### Antifungal drugs

All antifungal drugs were obtained from Sigma-Aldrich and Fisher Scientific. AmB, fluconazole, cycloheximide, nystatin dihydrate and itraconazole were reconstituted with water to appropriate concentrations. Ketoconazole was prepared in dimethyl sulfoxide. All stock dilutions were stored at –20°C for up to 2 months.

### Strains, media and growth conditions

The strains of *S. cerevisiae*, listed in Table 2, were grown at 30°C and maintained on yeast extract/peptone/dextrose medium (YPD). YPD and synthetic media (SD) were prepared as described by Rose *et al*. [51], YPD media containing G418 (200 µg/mL) was used for selection of strains with kanMX4 dominant drug-resistance markers. SD uracil-deficient medium was used for selection of strains with URA3 marker.

**Table 2:** Yeast strains and plasmids used in this study.

Strain	Genotype	Parental Strain	Source
BY4741	*MATa ho::KanMX his3 leu2 met15 ura3*		Yeast Knock-out (YKO) deletion collection
YJM789	*MATα ho::hisG lys2 cyh*		[8]
GY00	*MATα ho::hisG lys2 cyh ura3::KanMX*	YJM789	This study
GB01	*MATa ho::KanMX his3 leu2 met15 ura3 pdr5::URA3*	BY4741	This study
GY01	GY00, *pdr5::URA3*	YJM789	This study
GY02	GY01, *ura3::BPDR5*	YJM789	This study
GY03	GY02, *bpdr5::URA3*	YJM789	This study

### Transformation

Transformation of yeast with plasmid DNA was achieved according to the procedure previously described [52]. Bacterial transformation was performed using the calcium chloride procedure as described by Sambrook et al. [53].

### Strain construction

The strains GY01 (YJM789Δ*pdr5*) and GB01 (BY4741Δ*pdr5*) were constructed by replacing the *PDR5* gene from YJM789 and BY4741 parental strains using a URA3 marker, respectively. Correct integration of the deletion constructs and proper looping-out were confirmed by PCR analysis.

Strain GY02 was constructed by inserting BY4741 *PDR5* gene (*BPDR5*) into the *PDR5* locus of GY01. The transformants were selected on 5-FOA plates [54]. Because the viability of the mutants (GY02) in antifungal drugs might be influenced by 5-FOA, the *BPDR5* gene was disrupted in GY02 by targeted insertion with *URA3* as the selection marker to reconstruct the *pdr5* null mutants (GY03).

### Agar-plate drug-sensitivity assays

Fresh *S. cerevisiae* colonies were inoculated in 5 mL of YPD or SD selective medium, and grown overnight at 30°C. The cells were diluted to an OD_600_ of 0.2, and 4 µL was spotted with serial dilutions on solid medium containing drugs (AmB, fluconazole, itraconazole or ketoconazole) in the agar. The plates were incubated at 30°C for 48 h.

### Real-time drug-sensitivity assay

To test drug resistance, strains were grown overnight, diluted in 90 µL of rich medium (YPD), treated with 10 µL of water or a pharmacological compound, and then grown for 20 to 24 h. Samples were grown in a microplate spectrophotometer (OD 600) (model Uμ-MQX200R; Bio-T Instrument Inc.). Drug resistance was estimated from the optical density after incubating for 20 to 24 h (20 h for AmB and 24 h for azoles).

### Plasmids construction and transformation

The 5.18 kb *PDR5* fragment including 404 bp upstream and 224 bp downstream sequences was amplified from YJM789 or BY4741 genomic DNA (using primers Xba I_upper/Xba I_lower) and then inserted into the Xba I site of vector YEplac195, generating YEplac195-*YPDR5* or YEplac195-*BPDR5*. For the replacement of YJM789 *PDR5* transmembrane domains (TMDR1 or TMDR2) with the BY4741 *PDR5* TMDRs, 2.7KB (1–2764 bp) and 1.8 KB (2764–4535 bp) fragments of *PDR5* ORF which include BY4747 *PDR5* TMDR1 or TMDR2 encoding sequences were amplified by using primer combinations Sac I-upper/BstP I-lower and BstP I-upper/Sal I-lower, and then cloned into Sac I-BstP I and BstP I-Sal I digested YEplac195-*YPDR5*, respectively. All the above reconstructed plasmids were confirmed by colony PCR and DNA sequencing. Primer sequences used in this work (the underlined sequences indicate restriction sites, the restriction site of BstP I is inside the PCR product) are listed in Table 3.

**Table 3:** Primers used in *PDR5* plasmid construction.

Primer	Sequence
Xba I_upper	gctctagaCACGATTCAGCACCCTTTG
Xba I_lower	gctctagaACCGATGAGATAACCTAGAAAT
Sac I_ upper	ggtgagctcCACGATTCAGCACCCTTTG
BstP I_lower	GATTTATCACGGGGAATACCAT
BstP I_upper	TATCAATCCGTTGGCTTACTT
Sal I_lower	cgcgtcgacACCGATGAGATAACCTAGGAAT

### Construction of GFP-tagged yeast strains and fluorescence microscopy

Pdr5p-GFP protein fusions were constructed as previous described[55]. The 2.6kb GFP-kanMX6 cassette was amplified from pFA6a-GFP(S65T)-kanMX6 with a pair of chimeric primers: *TGGCTAGCAAGGGTACCTAAAAGGAATGGTAAACTCTCCAAGAAAGcttcgt* *acgCtgcaggtc* , *CGATGAGATAACCTAGAAATAAAATTCTCGGAATTCTTTCGGACcc* *aatacgcaaaccgcctct* . The PCR product was transformed into YJM789 WT and the transformants were selected on YPD plates containing G418. Integration of the GFP fusion at the *PDR5* locus was confirmed by PCR using combinations of primers flanking *PDR5* gene and GFP-kanMX6 cassette-specific primers. Fluorescence analysis was done using exponentially growing cells. Cells expressing Pdr5p-GFP fusion protein were examined by using fluorescence and differential interference contrast (DIC) microscopy (OLYMPUS BX51 microscope).

### RNA preparation and RT-PCR

To confirm *PDR5-1* (*YPDR5* TMDR1 + *BPDR5* TMDR2) and *PDR5-2* (*BPDR5* TMDR1 + *YPDR5* TMDR2) could express in BY4741Δ*pdr5* background, total RNA was prepared as described in an earlier study[56]. The cDNA was synthesized by PrimeScript™ RTase (Takara). Primer pairs (TGAGGTCTTCATCTCCTT/AAACCAACCTCTCCGTGT for *PDR5-1*, the PCR product length is 1111 bp, GCAAGACCTTCCTCTCCT/CAATTTCTCCGTAAGT ATCG for *PDR5-2*, the PCR product length is 1114 bp) were used to detect transcription of TMDR1 and TMDR2 constructs.

### Substitution rate analysis

To calculate evolutionary distance between two strains, we used PAML program to calculate the substitution rates of synonymous sites (*K_S_*) and nonsynonymous sites (*K_A_*) as previously described [57].

### Phylogenetic tree reconstruction

All sequenced *S. cerevisiae* strains and four *S. paradoxus* strains, one from each of four clades in this species [41], were used for tree reconstruction. *S. bayanus* sequence was used as outgroup. The combined TMDRs in each strain were aligned by Muscle [58] and tree was reconstructed by Clustalw [59]. The phylogenetic tree is shown in Figure S3.

## Supporting Information

Figure S1 Growth difference between YJM789Δpdr5::BPDR5 (GY02) and YJM789Δpdr5 (GY03) mutants in media containing fluconazole at 37°C. GY02(•) and GY03(-) were grown in YPD overnight at 30°C and reinoculated to OD600 = 0.1. 90 µl above media of strains were treated with 10 µl of water containing fluconazole (Final concentration is 20 µg/ml), and then grown for 24 h at 37°C in a microplate spectrophotometer (model Uμ-MQX200R; Bio-T Instrument Inc.). OD600 value was sampled every 20 minutes.
(0.03 MB DOC)

Figure S2 Multiple pathogenic S. cerevisiae strains have divergent sequence in PDR5 gene. SNP distributions for PDR5 gene from all 63 strains were generated from http://gbrowse.princeton.edu/cgi-bin/gbrowse/yeast_strains_snps/ (YSB, Yeast SNPs Browser [39]). Eleven strains were shown in this figure, and the pathogenic strains were highlighted in red rectangular. Among all 63 strains, five pathogenic strains (including YJM145 that is isogenic to YJM789), which were from different clinical isolation [7], display dramatic sequence divergence in PDR5 gene, while the remaining clinical strains (YJM421 was shown as an example) show only a few SNPs. In contrast, none of the non-clinical strains (five were shown as example) show dramatic sequence divergence in PDR5 gene.
(0.04 MB DOC)

Figure S3 Phylogenetic tree for the combined two transmembrane domain regions (TMDR) in PDR5 (YOR153W) gene. All sequenced S. cerevisiae strains and four S. paradoxus strains (marked in green), one from each of four clades in this species [41], were used for tree reconstruction. S. bayanus sequence was used as outgroup. The combined TMDRs in each strain were aligned by Muscle [59] and tree was reconstructed by Clustalw [60]. Bootstrap values are shown on the tree. As indicated in the figure, the PDR5 gene in YJM789 (indicated by red arrow) is clearly an outgroup in all S. cerevisiae strains, which is not true for whole genome sequence comparison(3). The PDR5 gene in YJM789 is also very divergent from all S. paradoxus strains (including all sequenced strains, only four were shown here). For S. mikatae, S. kudriavzevii, S. cariocanus, as they either are more divergent than S. paradoxus from S. cerevisiae, or their PDR5 gene sequences are not available, we didn't include them in the phylogenetic analysis.
(0.05 MB DOC)

## References

[1] Groll AH, Tragiannidis A (2009). Recent advances in antifungal prevention and treatment.. Semin Hematol.

[2] Sanglard D, Coste A, Ferrari S (2009). Antifungal drug resistance mechanisms in fungal pathogens from the perspective of transcriptional gene regulation.. FEMS Yeast Res.

[3] Andersson DI, Hughes D (2010). Antibiotic resistance and its cost: is it possible to reverse resistance?. Nat Rev Microbiol.

[4] Goffeau A (2008). Drug resistance: The fight against fungi.. Nature.

[5] White TC, Marr KA, Bowden RA (1998). Clinical, cellular, and molecular factors that contribute to antifungal drug resistance.. Clin Microbiol Rev.

[6] Tawfik OW, Papasian CJ, Dixon AY, Potter LM (1989). *Saccharomyces cerevisiae* pneumonia in a patient with acquired immune-deficiency syndrome.. J Clin Microbiol.

[7] Mccusker JH, Clemons KV, Stevens DA, Davis RW (1994). Genetic-characterization of pathogenic *Saccharomyces cerevisiae* isolates.. Genetics.

[8] Steinmetz LM, Sinha H, Richards DR, Spiegelman JI, Oefner PJ (2002). Dissecting the architecture of a quantitative trait locus in yeast.. Nature.

[9] Mccusker JH, Clemons KV, Stevens DA, Davis RW (1994). *Saccharomyces cerevisiae* virulence phenotype as determined with Cd-1 mice is associated with the ability to grow at 42°C and form pseudohyphae.. Infect Immun.

[10] Wei W, McCusker JH, Hyman RW, Jones T, Ning Y (2007). Genome sequencing and comparative analysis of *Saccharomyces cerevisiae* strain YJM789.. Proc Natl Acad Sci USA.

[11] Tamm M (1999). The lung in the immunocompromised patient: Infectious complications part 2.. Respiration.

[12] Ruhnke M (2004). Mucosal and systemic fungal infections in patients with AIDS - Prophylaxis and treatment.. Drugs.

[13] Mamnun YM, Schuller C, Kuchler K (2004). Expression regulation of the yeast PDR5 ATP-binding cassette (ABC) transporter suggests a role in cellular detoxification during the exponential growth phase.. FEBS Letters.

[14] Kontoyiannis DP (2000). Efflux-mediated resistance to fluconazole could be modulated by sterol homeostasis in *Saccharomyces cerevisiae*.. J Antimicrob Chemoth.

[15] Chen XJ (2001). Activity of the Kluyveromyces lactis Pdr5 multidrug transporter is modulated by the sit4 protein phosphatase.. J Bacteriol.

[16] Seret ML, Diffels JF, Goffeau A, Baret PV (2009). Combined phylogeny and neighborhood analysis of the evolution of the ABC transporters conferring multiple drug resistance in hemiascomycete yeasts.. BMC Genomics.

[17] Leppert G, Mcdevitt R, Falco SC, Vandyk TK, Ficke MB (1990). Cloning by gene Amplification of 2 loci conferring multiple-drug resistance in *Saccharomyces*.. Genetics.

[18] Meyers S, Schauer W, Balzi E, Wagner M, Goffeau A (1992). Interaction of the yeast pleiotropic drug-resistance gene PDR1 and PDR5.. Curr Genet.

[19] Hirata D, Yano K, Miyahara K, Miyakawa T (1994). *Saccharomyces cerevisiae* Ydr1, which encodes a member of the ATP-binding cassette (ABC) superfamily, is required for multidrug-resistance.. Curr Genet.

[20] Shahi P, Gulshan K, Moye-Rowley WS (2007). Negative transcriptional regulation of multidrug resistance gene expression by an Hsp70 protein.. J Biol Chem.

[21] Ernst R, Klemm R, Schmitt L, Kuchler K (2005). Yeast ATP-binding cassette transporters: Cellular cleaning pumps.. Methods Enzymol.

[22] Holland IB, Blight MA (1999). ABC-ATPases, adaptable energy generators fuelling transmembrane movement of a variety of molecules organisms from bacteria to humans.. J Mol Biol.

[23] Tutulan-Cunita AC, Mikoshi M, Mizunuma M, Hirata D, Miyakawa T (2005). Mutational analysis of the yeast multidrug resistance ABC transporter Pdr5p with altered drug specificity.. Genes Cells.

[24] Balzi E, Wang M, Leterme S, Vandyck L, Goffeau A (1994). Pdr5p, a novel yeast multidrug-resistance conferring transporter controlled by the transcription regulator PDR1.. J Biol Chem.

[25] Dismukes WE (2000). Introduction to antifungal drugs.. Clin Infect Dis.

[26] Decottignies A, Goffeau A (1997). Complete inventory of the yeast ABC proteins.. Nat Genet.

[27] Groeschke J, Solassol I, Bressolle F, Pinguet F (2006). Stability of amphotericin B and nystatin in antifungal mouthrinses containing sodium hydrogen carbonate.. J Pharmaceut Biomed.

[28] Chesson P (2000). Mechanisms of maintenance of species diversity.. Annu Rev Ecol Syst.

[29] Cooper VS, Lenski RE (2000). The population genetics of ecological specialization in evolving Escherichia coli populations.. Nature.

[30] Futuyma DJ, Moreno G (1988). The evolution of ecological specialization.. Annu Rev Ecol Syst.

[31] Levene H (1953). Genetic equilibrium when more than one ecological niche is available.. Am Nat.

[32] Li WH, Saunders MA (2005). The chimpanzee and us.. Nature.

[33] Olson MV (1999). When less is more: gene loss as an engine of evolutionary change.. Am J Hum Genet.

[34] Aminetzach YT, Macpherson JM, Petrov DA (2005). Pesticide resistance via transposition-mediated adaptive gene truncation in Drosophila.. Science.

[35] Greenberg AJ, Moran JR, Coyne JA, Wu CI (2003). Ecological adaptation during incipient speciation revealed by precise gene replacement.. Science.

[36] Hittinger CT, Rokas A, Carroll SB (2004). Parallel inactivation of multiple GAL pathway genes and ecological diversification in yeasts.. Proc Natl Acad Sci USA.

[37] Wang XX, Grus WE, Zhang JZ (2006). Gene losses during human origins.. PLoS Biol.

[38] Winzeler EA, Richards DR, Conway AR, Goldstein AL, Kalman S (1998). Direct allelic variation scanning of the yeast genome.. Science.

[39] Schacherer J, Shapiro JA, Ruderfer DM, Kruglyak L (2009). Comprehensive polymorphism survey elucidates population structure of *Saccharomyces cerevisiae*.. Nature.

[40] Stroup JS, Stephens JR, Baker DL, Lad M (2007). Amphotericin B formulation confusion and mortality in an HIV-Seropositive patient.. Hosp Pharm.

[41] Liti G, Carter DM, Moses AM, Warringer J, Parts L (2009). Population genomics of domestic and wild yeasts.. Nature.

[42] Ghannoum MA, Rice LB (1999). Antifungal agents: Mode of action, mechanisms of resistance, and correlation of these mechanisms with bacterial resistance.. Clin Microbiol Rev.

[43] Chu P, Sadullah S (2009). The current role of amphotericin B lipid complex in managing systemic fungal infections.. Curr Med Res Opin.

[44] Decottignies A, Grant AM, Nichols JW, de Wet H, McIntosh DB (1998). ATPase and multidrug transport activities of the overexpressed yeast ABC protein Yor1p.. J Biol Chem.

[45] Mukhopadhyay K, Prasad T, Saini P, Pucadyil TJ, Chattopadhyay A (2004). Membrane sphingolipid-ergosterol interactions are important determinants of multidrug resistance in *Candida albicans*.. Antimicrob Agents Chemother.

[46] Kihara A, Igarashi Y (2004). Cross talk between sphingolipids and glycerophospholipids in the establishment of plasma membrane asymmetry.. Mol Biol Cell.

[47] Golin J, Ambudkar SV, May L (2007). The yeast Pdr5p multidrug transporter: how does it recognize so many substrates?. Biochem Biophys Res Commun.

[48] Young LY, Hull CM, Heitman J (2003). Disruption of ergosterol biosynthesis confers resistance to amphotericin B in *Candida lusitaniae*.. Antimicrob Agents Chemother.

[49] Vandeputte P, Tronchin G, Larcher G, Ernoult E, Berges T (2008). A nonsense mutation in the ERG6 gene leads to reduced susceptibility to polyenes in a clinical isolate of *Candida glabrata*.. Antimicrob Agents Chemother.

[50] Mancera E, Bourgon R, Brozzi A, Huber W, Steinmetz LM (2008). High-resolution mapping of meiotic crossovers and non-crossovers in yeast.. Nature.

[51] Rose MD, Winston FM, Hieter P (1990).

[52] Hinnen A, Hicks JB, Fink GR (1978). Transformation of yeast.. Proc Natl Acad Sci USA.

[53] Sambrook J, Russell DW (2006).

[54] Boeke JD, Trueheart J, Natsoulis G, Fink GR (1987). 5-fluoroorotic acid as a selective agent in yeast molecular genetics.. Methods in Enzymology.

[55] de Thozee CP, Cronin S, Goj A, Golin J, Ghislain M (2007). Subcellular trafficking of the yeast plasma membrane ABC transporter, PDR5, is impaired by a mutation in the N-terminal nucleotide-binding fold.. Mol Microbiol.

[56] Schmitt ME, Brown TA, Trumpower BL (1990). A rapid and simple method for preparation of RNA from *Saccharomyces cerevisiae*.. Nucleic Acids Res.

[57] Yang ZH (2007). PAML 4: Phylogenetic analysis by maximum likelihood.. Mol Biol Evol.

[58] Edgar RC (2004). MUSCLE: multiple sequence alignment with high accuracy and high throughput.. Nucleic Acids Res.

[59] Thompson JD, Higgins DG, Gibson TJ (1994). ClustalW - improving the sensitivity of progressive multiple sequence alignment through sequence weighting, position-specific gap penalties and weight matrix choice.. Nucleic Acids Res.

[60] Fitzpatrick DA, Logue ME, Stajich JE, Butler G (2006). A fungal phylogeny based on 42 complete genomes derived from supertree and combined gene analysis.. BMC Evol Biol.

