# Supplementary material for: Antagonistic Changes in Sensitivity to Antifungal Drugs by Mutations of an Important ABC Transporter Gene in a Fungal Pathogen
Source: PLoS One. 2010 Jun 25;5(6):e11309. doi: 10.1371/journal.pone.0011309 (PMC2892482; doi:10.1371/journal.pone.0011309)
Supplement: Supplementary file 1 [file pone.0011309.s001.doc]

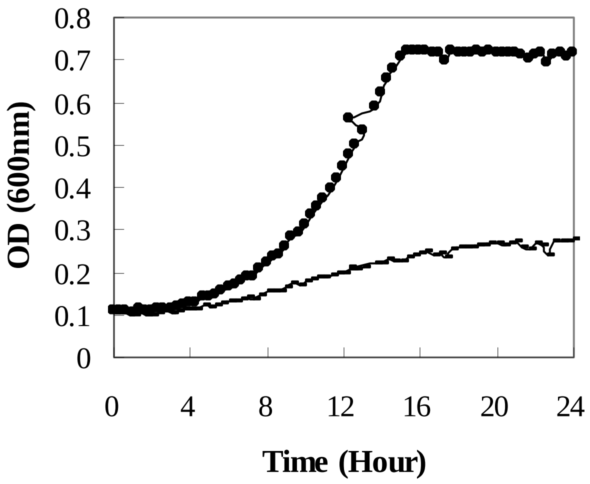
0.8
0.7
0.6
0.5
0.4
0.3
0.2
0.1
0
OD (600nm)
0
4
8
12
16
20
24
Time (Hour)
