# Supplementary material for: Antagonistic Changes in Sensitivity to Antifungal Drugs by Mutations of an Important ABC Transporter Gene in a Fungal Pathogen
Source: PLoS One. 2010 Jun 25;5(6):e11309. doi: 10.1371/journal.pone.0011309 (PMC2892482; doi:10.1371/journal.pone.0011309)
Supplement: Supplementary file 2 [file pone.0011309.s002.doc]

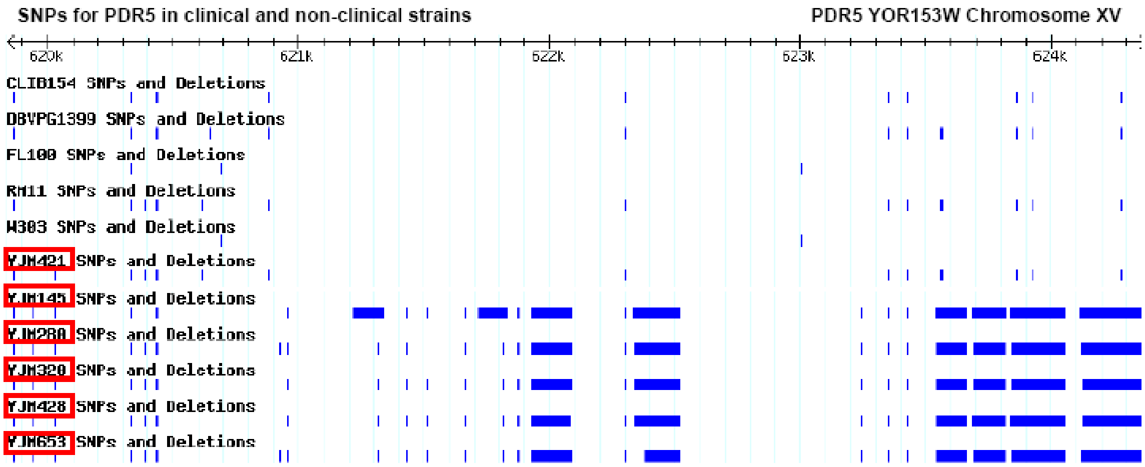
SNPs for PDR5 in clinical and non-clinical strains
PDR5 YOR153W Chromosome XV
620k
621k
622k
623k
624k
CLIB154 SNPs and Deletions
DBVPG1399 SNPs and Deletions
FL100 SNPs and Deletions
RM11 SNPs and Deletions
W303 SNPs and Deletions
YJM421 SNPs and Deletions
YJM145 SNPs and Deletions
YJM280 SNPs and Deletions
YJM320 SNPs and Deletions
YJM428 SNPs and Deletions
YJM653 SNPs and Deletions
