# Supplementary material for: Antagonistic Changes in Sensitivity to Antifungal Drugs by Mutations of an Important ABC Transporter Gene in a Fungal Pathogen
Source: PLoS One. 2010 Jun 25;5(6):e11309. doi: 10.1371/journal.pone.0011309 (PMC2892482; doi:10.1371/journal.pone.0011309)
Supplement: Supplementary file 3 [file pone.0011309.s003.doc]

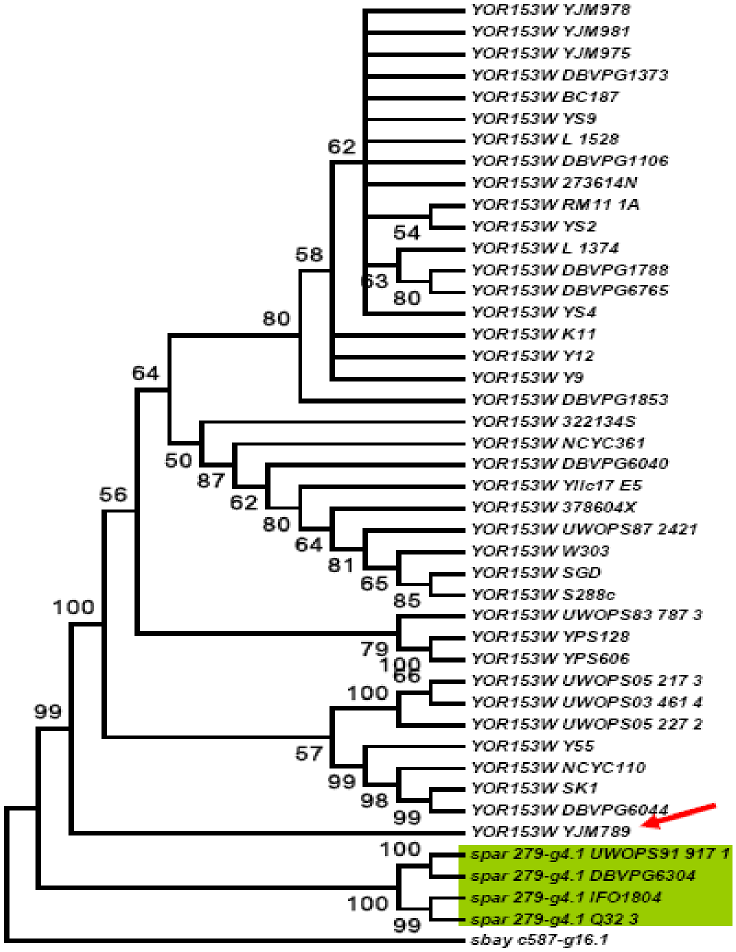
YOR153W YJM978
YOR153W YJM981
YOR153W YJM975
YOR153W DBVPG1373
YOR153W BC187
YOR153W YS9
YOR153W L 1528
YOR153W DBVPG1106
YOR153W 273614N
YOR153W RM11 1A
YOR153W YS2
YOR153W L 1374
YOR153W DBVPG1788
YOR153W DBVPG6765
YOR153W YS4
YOR153W K11
YOR153W Y12
YOR153W Y9
YOR153W DBVPG1853
YOR153W 322134S
YOR153W NCYC361
YOR153W DBVPG6040
YOR153W YIIc17 E5
YOR153W 378604X
YOR153W UWOPS87 2421
YOR153W W303
YOR153W SGD
YOR153W S288c
YOR153W UWOPS83 787 3
YOR153W YPS128
YOR153W YPS606
YOR153W UWOPS05 217 3
YOR153W UWOPS03 461 4
YOR153W UWOPS05 227 2
YOR153W Y55
YOR153W NCYC110
YOR153W SK1
YOR153W DBVPG6044
YOR153W YJM789
spar 279-g4.1 UWOPS91 917 1
spar 279-g4.1 DBVPG6304
spar 279-g4.1 IFO1804
spar 279-g4.1 Q32 3
sbay c587-g16.1
